# Public awareness of mental illness: Mental health literacy or concept creep?

**DOI:** 10.1177/10398562241292202

**Published:** 2024-10-14

**Authors:** Nick Haslam, Jesse SY Tse

**Affiliations:** Melbourne School of Psychological Sciences, 2281 University of Melbourne, Parkville, VIC, Australia

**Keywords:** concept creep, mental health literacy, overdiagnosis, social stigma

## Abstract

Rising awareness of mental illness has increased the public’s mental health literacy, with positive implications for help-seeking and destigmatization. We argue that it has also enlarged the public’s concept of mental illness. People have become better at recognizing the presence of mental illness but may have become worse at recognizing its absence. This conceptual expansion fosters unwarranted self-diagnosis, the pathologization of ordinary distress, and unnecessary treatment. It is incumbent on mental health professionals to promote accurate knowledge of mental illness and push back against overly expansive concepts of it.

Accurate knowledge about mental illness should help people respond to it effectively and compassionately. This insight is embodied in the influential concept of “mental health literacy” (MHL), introduced by Australian researchers in 1997.^1^ Originally defined as “knowledge and beliefs about mental disorders which aid their recognition, management, or prevention,” MHL is often assessed by the capacity to correctly identify mental disorders from vignette descriptions. Analysis of historical trends indicates that the public’s MHL may be rising,^2,3^ perhaps driven by increased awareness of mental illness.

Paralleling the rise in MHL, there has been an enlargement of the public’s concept of mental illness. Studies demonstrate that mental health–related concepts have broadened their meanings—a trend known as “concept creep”—so that they now encompass a wider range of phenomena than in earlier decades.^4^ Laypeople now commonly hold expansive concepts, one recent study finding that American adults judged 11 non-DSM-5 conditions to be mental disorders, including persistent grief, excessive jealousy, problematic internet gaming, and low self-esteem.^5^ This cultural shift mirrors inflationary trends in psychiatric classification which, critics argue, pathologize everyday distress and promote overdiagnosis.^6,7^ We contend that it is vital to distinguish the expansiveness of people’s concepts of mental illness from the accuracy of their knowledge about it. A broad concept need not be a valid one. MHL and broad concepts of mental illness both have some benefits, but overly broad concepts also have some adverse implications.

## The relationship between mental health literacy and concept breadth

The distinction between MHL and concept breadth can be understood through the lens of signal detection theory. When judging whether a signal is present, four possibilities exist: A “hit” when the signal is correctly judged to be present, a “correct rejection” when it is correctly judged to be absent, a “false alarm” when it is incorrectly judged present, and a “miss” when it is incorrectly judged absent. Signal detection analysis models these judgments using two parameters. *Sensitivity* is the level of accuracy (i.e., the degree to which hits and correct rejections exceed misses and false alarms) and *bias* is the general tendency to judge the signal to be present (or absent). A judge with a liberal bias has a low threshold for detecting the signal and will therefore score many hits but also make many false alarms. A judge with a conservative bias will make fewer false alarms but also score fewer hits.

MHL is akin to sensitivity, capturing how accurately a person identifies mental illness. Concept breadth, in contrast, is akin to bias. People with expansive concepts frequently judge mental illness to be present, leading to many hits but also many false alarms (i.e., over-diagnoses), whereas those with narrow concepts rarely judge it present, leading to many correct rejections but also many missed diagnoses. Sensitivity and bias are independent parameters, so every combination of high and low levels of MHL and concept breadth can occur.

Several implications follow from this analysis. First, holding a broad concept of mental illness is no guarantee of accuracy. In earlier decades, when laypeople’s concepts of mental illness were overly narrow,^8^ people with broader concepts may have had greater MHL, but in the present time broad concepts can be inaccurate. Second, any factor that broadens concepts of mental illness will increase diagnostic false positives (i.e., reduce “specificity”), unless counteracted by a rise in MHL. Third, although efforts to boost MHL by raising mental health awareness may enhance accurate knowledge, they may also broaden concepts of mental illness. Increasing awareness of diagnostic concepts, for example, may induce bias rather than accuracy if people adopt low thresholds for applying them.

It is not surprising that members of the public might understand mental illness in ways that are both accurate and overly expansive. Psychopathology typically falls on a spectrum and diagnosis usually relies on a set of imprecise severity or frequency criteria and subjective judgments of clinically significant distress or impairment. Laypeople may have accurate knowledge of the clinical features of a mental illness, and consequently a good capacity to recognize prototypical cases, but also have a poorly calibrated sense of the severity threshold required for a diagnosis. In this way, high MHL can co-exist with expansive concepts of mental illness.

## Evidence and implications

Recent research supports our contention that holding broad concepts of mental illness should be distinguished from MHL and that they have important implications. Supporting the distinctness of the factors, new psychometric scales for measuring concept breadth^9^ show that it is barely correlated with MHL, consistent with them being akin to independent accuracy and bias parameters. Supporting the role of broad concepts in overdiagnosis, research using these scales shows that people holding broad concepts are more likely to self-diagnose in the absence of a professional diagnosis than people with narrower concepts who are experiencing the same levels of distress.^10^ Groups who are especially likely to self-diagnose—young adults and political progressives—were also most likely to hold broad concepts. Holding broad concepts of mental illness has some positive implications, such as greater willingness to seek help and lesser stigma,^9^ but studies such as these suggest it also carries risks.

Four overlapping risks stand out. First, because broad concepts of mental illness foster self-diagnosis, they are partly responsible for its problematic consequences. One study found that young adults who self-labelled with depression believed they had less control over their illness and coped less effectively with it than equally depressed people who did not self-diagnose, implying that broad concepts of mental illness that encourage self-labelling may undermine recovery.^11^ Second, it has recently been argued that self-diagnosis can contribute to the development of mental illness via self-fulfilling processes.^12^ For example, identifying as clinically anxious can lead people to avoid threatening situations, which can deepen and entrench their anxiety. This finding aligns with evidence that inducing people to hold a broad concept of trauma leads them to develop more posttraumatic symptoms following an unpleasant experience.^13^ Third, unwarranted self-diagnosis can set in motion unwarranted formal diagnosis. Patients may seek ratification of their self-diagnosis from a mental health professional, who may oblige out of a well-intentioned desire to secure treatment or accommodations for the patient.^14^ Fourth, broad concepts of mental illness are especially likely to bring people with relatively mild problems into treatment, and such people may be least likely to benefit from it. Indeed, recent research found that patients experiencing relatively mild anxiety or depression symptoms who accessed mental health services through Australia’s Better Access initiative were considerably more likely to get worse than to improve.^15^ Broad concepts of mental illness may therefore expose people to adverse iatrogenic effects.

Increased public awareness of mental illness is a goal few would oppose. However, we should be concerned if, in addition to disseminating accurate knowledge, it is fostering expansive concepts of illness that lead people to pathologize everyday, subclinical distress. Rising awareness can be a double-edged sword, improving our MHL but also making our mental health worse by promoting identification with self-limiting and potentially self-fulfilling diagnoses.^11^ Broad concepts of mental illness may be contributing to a trend of overdiagnosis that has potentially dire implications. It is vital that psychiatrists and other mental health professionals find ways to boost accurate knowledge about mental illness without simultaneously inflating it, and that they push back against overly expansive concepts.
